# Supplementary figures and images for: Genetics of cocaine and methamphetamine consumption and preference in Drosophila melanogaster
Source: PLoS Genet. 2019 May 20;15(5):e1007834. doi: 10.1371/journal.pgen.1007834 (PMC6527214; doi:10.1371/journal.pgen.1007834)

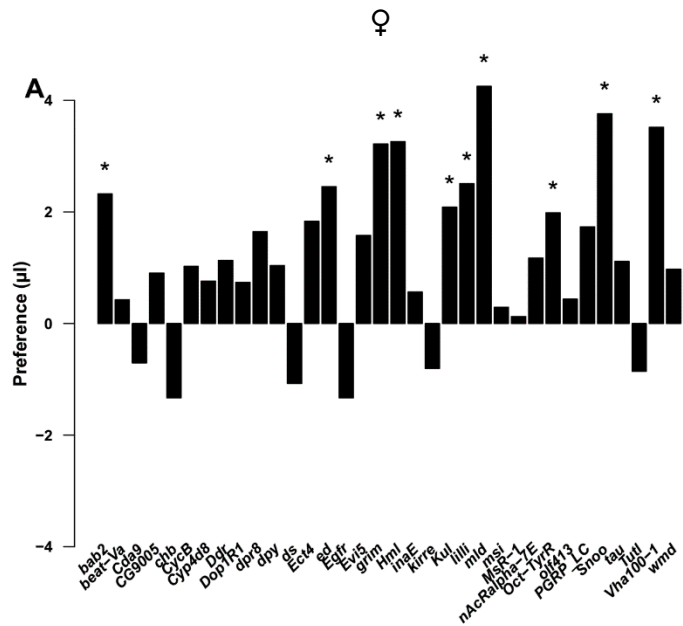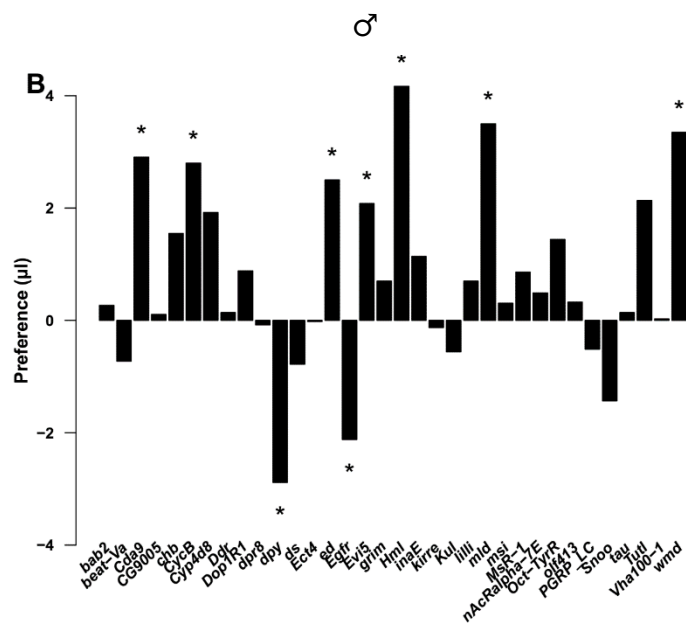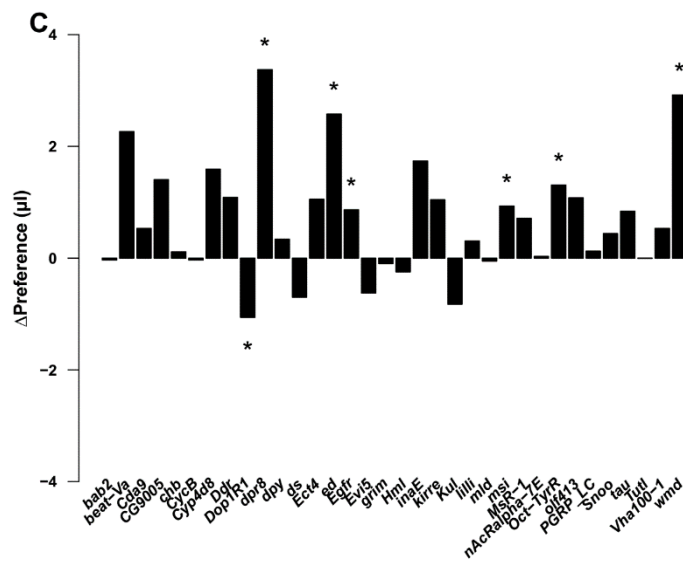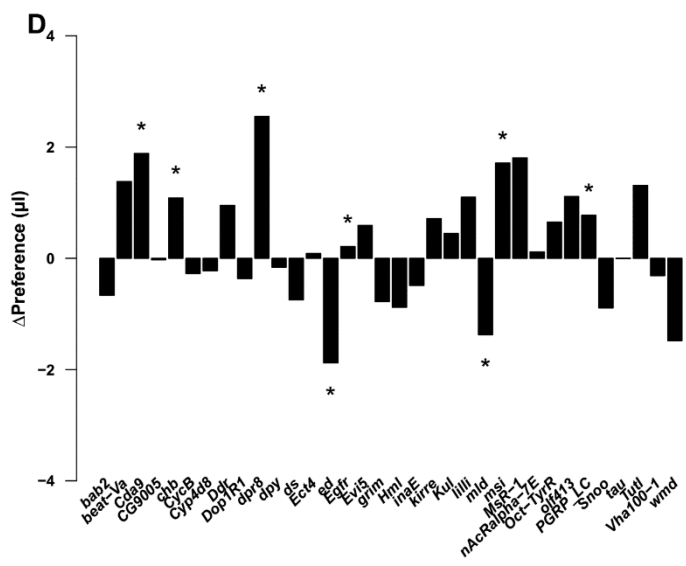

Supplement: S2 Fig — (A) Cocaine preference, females. (B) Cocaine preference, males. (C) Change in cocaine preference between third and first exposures, females. (D) Change in cocaine preference between third and first exposures, males. Asterisks represent significant L×S terms (A, B) or significant L×S×E terms from the full ANOVA models. Exact P-values are given in S10 Table. (PDF) [file pgen.1007834.s014.pdf]

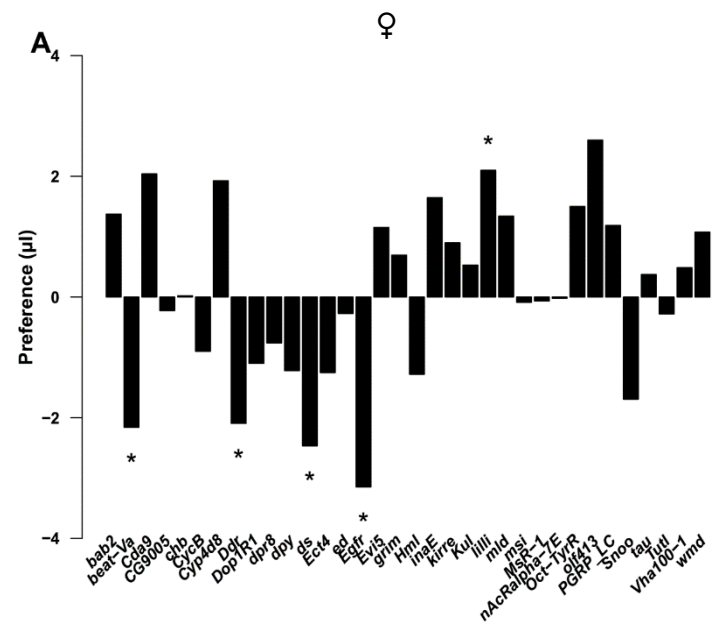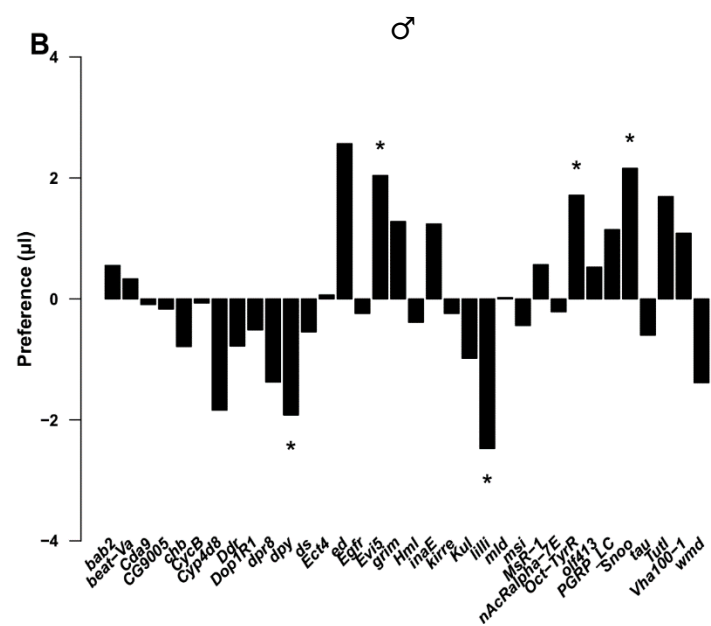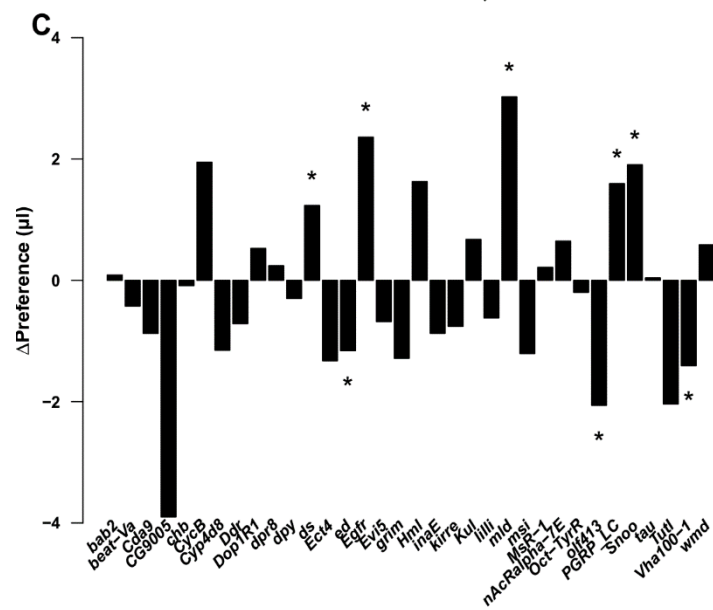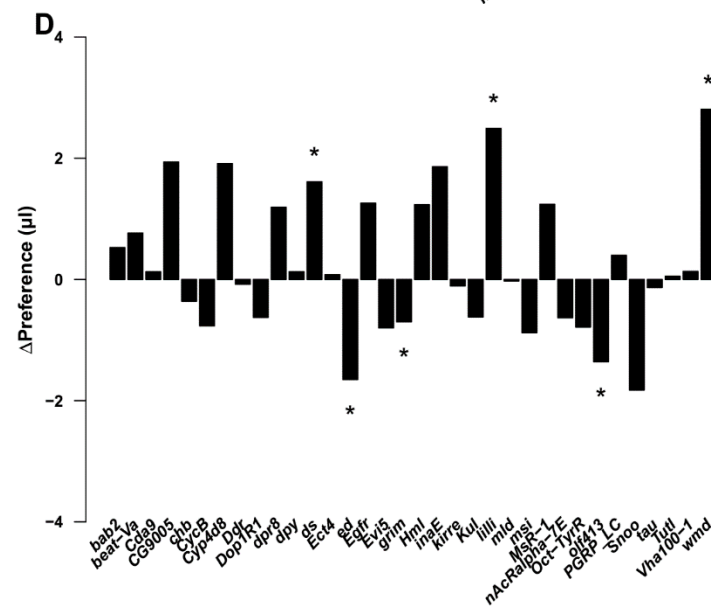

Supplement: S3 Fig — (A) Methamphetamine preference, females. (B) Methamphetamine preference, males. (C) Change in methamphetamine preference between third and first exposures, females. (D) Change in methamphetamine preference between third and first exposures, males. Asterisks represent significant L×S terms (A, B) or significant L×S×E terms from the full ANOVA models. Exact P-values are given in S11 Table. (PDF) [file pgen.1007834.s015.pdf]
