# Supplementary material for: Genetics of cocaine and methamphetamine consumption and preference in Drosophila melanogaster
Source: PLoS Genet. 2019 May 20;15(5):e1007834. doi: 10.1371/journal.pgen.1007834 (PMC6527214; doi:10.1371/journal.pgen.1007834)
Supplement: S4 Fig — Red: P < 0.0001; orange: P < 0.001; yellow: P < 0.01; green: P < 0.05; white: P > 0.05. (PDF) [file pgen.1007834.s016.pdf]

| | | | *elav-GAL4* | | | | *repo-GAL4* | | | |
|---|---|---|---|---|---|---|---|---|---|---|
| | | | Consumption | Δ Consumption | Preference | Δ Preference | Consumption | Δ Consumption | Preference | Δ Preference |
| *Dop1R1* | Cocaine | ♀ | | | | | | | | |
| | | ♂ | | | | | | | | |
| | Methamphetamine | ♀ | | | | | | | | |
| | | ♂ | | | | | | | | |
| *Ect4* | Cocaine | ♀ | | | | | | | | |
| | | ♂ | | | | | | | | |
| | Methamphetamine | ♀ | | | | | | | | |
| | | ♂ | | | | | | | | |
| *ed* | Cocaine | ♀ | | | | | | | | |
| | | ♂ | | | | | | | | |
| | Methamphetamine | ♀ | | | | | | | | |
| | | ♂ | | | | | | | | |
| *mld* | Cocaine | ♀ | | | | | | | | |
| | | ♂ | | | | | | | | |
| | Methamphetamine | ♀ | | | | | | | | |
| | | ♂ | | | | | | | | |
| *msi* | Cocaine | ♀ | | | | | | | | |
| | | ♂ | | | | | | | | |
| | Methamphetamine | ♀ | | | | | | | | |
| | | ♂ | | | | | | | | |
| *Oct-TyrR* | Cocaine | ♀ | | | | | | | | |
| | | ♂ | | | | | | | | |
| | Methamphetamine | ♀ | | | | | | | | |
| | | ♂ | | | | | | | | |
| *olf413* | Cocaine | ♀ | | | | | | | | |
| | | ♂ | | | | | | | | |
| | Methamphetamine | ♀ | | | | | | | | |
| | | ♂ | | | | | | | | |
| *Snoo* | Cocaine | ♀ | | | | | | | | |
| | | ♂ | | | | | | | | |
| | Methamphetamine | ♀ | | | | | | | | |
| | | ♂ | | | | | | | | |
| *Vha100-1* | Cocaine | ♀ | | | | | | | | |
| | | ♂ | | | | | | | | |
| | Methamphetamine | ♀ | | | | | | | | |
| | | ♂ | | | | | | | | |
| *wmd* | Cocaine | ♀ | | | | | | | | |
| | | ♂ | | | | | | | | |
| | Methamphetamine | ♀ | | | | | | | | |
| | | ♂ | | | | | | | | |
