# Supplementary material for: Genetics of cocaine and methamphetamine consumption and preference in Drosophila melanogaster
Source: PLoS Genet. 2019 May 20;15(5):e1007834. doi: 10.1371/journal.pgen.1007834 (PMC6527214; doi:10.1371/journal.pgen.1007834)
Supplement: S5 Fig — Red: P < 0.0001; orange: P < 0.001; yellow: P < 0.01; green: P < 0.05; white: P > 0.05. (PDF) [file pgen.1007834.s017.pdf]

			MB-GAL4				*TH-GAL4*			
			Consumption	Δ Consumption	Preference	Δ Preference	Consumption	Δ Consumption	Preference	Δ Preference
Dop1R1	**Cocaine**	♀								
		♂								
	**Methamphetamine**	♀								
		♂								
ed	**Cocaine**	♀								
		♂								
	**Methamphetamine**	♀								
		♂								
msi	**Cocaine**	♀								
		♂								
	**Methamphetamine**	♀								
		♂								
Snoo	**Cocaine**	♀								
		♂								
	**Methamphetamine**	♀								
		♂								
